# The breaking and making of healthy adult human skeletal muscle in vivo

**DOI:** 10.1186/s13395-017-0142-x

**Published:** 2017-11-07

**Authors:** Abigail L. Mackey, Michael Kjaer

**Affiliations:** 1 Institute of Sports Medicine Copenhagen, Department of Orthopaedic Surgery M, Bispebjerg Hospital, Copenhagen, Denmark; 2 Center for Healthy Aging, Department of Biomedical Sciences, Faculty of Health and Medical Sciences, University of Copenhagen, Copenhagen, Denmark; 3 Center for Healthy Aging, Faculty of Health and Medical Sciences, University of Copenhagen, Copenhagen, Denmark

**Keywords:** Basement membrane, Human myogenesis, Necrosis, Satellite cells

## Abstract

**Background:**

While muscle regeneration has been extensively studied in animal and cell culture models, in vivo myogenesis in adult human skeletal muscle has not been investigated in detail.

**Methods:**

Using forced lengthening contractions induced by electrical stimulation, we induced myofibre injury in young healthy males. Muscle biopsies were collected from the injured leg 7 and 30 days after muscle injury and from the uninjured leg as a control. Immuno-stained single muscle fibres and muscle cross sections were studied by wide-field and confocal microscopy. Samples were also studied at the ultra-structural level by electron microscopy.

**Results:**

Microscopy of single muscle fibres in 3 dimensions revealed a repeating pattern of necrotic and regenerating zones along the length of the same myofibre, characterised by extensive macrophage infiltration alongside differentiating myogenic progenitor cells and myotubes: the hallmarks of myogenesis. The myofibre basement membrane was preserved during these processes and interestingly was seen at a later stage as a second basement membrane surrounding the regenerating fibres.

**Conclusions:**

This is the first work to document in vivo myogenesis in humans in detail and highlights the importance of the basement membrane in the process of regeneration. In addition, it provides insight into parallels between the regeneration of adult skeletal muscle in mouse and man, confirming that this model may be a useful tool in investigating myofibre and matrix formation, as well as specific cell types, during regeneration from the perspective of human muscle.

## Background

The common purpose of studies investigating muscle regeneration is to contribute to the improved clinical treatment of conditions such as muscle disease, volumetric muscle loss and sarcopenia, as well as muscle injury itself, in humans. However, the study of muscle regeneration is largely based on animal studies, taking advantage of the possibilities for invasive interventions and where whole muscles can be excised for analyses. These mostly include focal muscle injury [1, 2], or various types of toxin injection into the muscle, as recently studied in detail [3]. The clear strength of animal models is the possibility for molecular manipulation, for example as exploited to demonstrate the absolute necessity of satellite cells for the regeneration of murine skeletal muscle following injury [4, 5]. Studying myogenesis in vitro has also proved to be a valuable tool, for example in determining intrinsic cell properties and mechanisms involved in the contribution of other cell types to muscle repair. However, similarities between these models and how myogenesis actually occurs in mature human muscle, in vivo, have to our knowledge never been investigated. Traumatic human skeletal muscle damage occurs in the form of either strain muscle rupture which occurs at the myotendinous junction and does not allow for study of muscle cell injury or contusion muscle damage where it is not possible in a systematic standardised way to study regeneration. Human models of muscle damage are thus usually exercise-based and consist of voluntary lengthening (eccentric) muscle contractions, which rarely lead to myofibre necrosis, despite indirect indications of muscle damage such as delayed onset muscle soreness and increased circulating levels of creatine kinase. While these pseudomarkers of muscle damage are common and can reach substantial levels, studies of muscle biopsies from such studies only occasionally report evidence of myofibre necrosis [6–8], suggesting a process of remodelling of the muscle and its associated extracellular matrix rather than true regeneration, as recently discussed [9, 10]. In contrast to this, the use of neuromuscular electrical stimulation in conjunction with forced lengthening contractions has been shown to be effective in inducing myofibre necrosis in human skeletal muscle, with concomitant proliferation of satellite cells and infiltration of macrophages [11]. We recently confirmed a large activation and proliferation of the satellite cell pool with this model and extended the time course of study to 30 days, where a large proportion of fibres demonstrated neonatal myosin expression [12], indicating the potential of this injury model to induce large-scale muscle regeneration, although how alike this process is to rodent muscle regeneration has rarely been addressed in the context of healthy human adult muscle.

While the different forms of muscle injury are associated with different repair trajectories, the processes of muscle regeneration share many similarities. In brief, damaged tissue is removed by recruited macrophages while myogenesis begins. The main stages of myogenesis in mature skeletal muscle include the appearance of myoblasts at the fibre periphery, which eventually fuse with each other to form myotubes. These myotubes enlarge and mature over time to restore the muscle to its pre-injury state, as reviewed elsewhere [9, 13]. Depending on the model of injury, as well as affecting the entire myofibre, necrosis can also occur segmentally, affecting only part of the myofibre [1, 2]. One of the pervading features reported to be required for successful regeneration following necrosis is the preservation of the muscle fibre basement membrane [14]. A recurring observation from electron microscopy studies is that of empty basement membrane tubes, present after fibre necrosis, in both animal muscle [14] and human dystrophic muscle [15]. Furthermore, there are reports of the appearance of a new basement membrane surrounding the regenerated myotubes, but within the “original” basement membrane which at this stage demonstrates a pleated form [16–18] and is thought to be removed gradually over time. Together, this suggests that the basement membrane is an important scaffold for myogenesis but at some point becomes redundant as the newly formed myofibres form their own basement membrane. The consequence of the formation of new myofibres, with a new sarcolemma and basement membrane, is a completely reconstructed satellite cell niche, which could be beneficial in certain situations. It is not known however if this occurs in human skeletal muscle in the adult state.

The aim of this study was to investigate the in vivo necrosis and regeneration of healthy adult human skeletal muscle with focus on stages of myogenesis and the myofibre basement membrane.

## Methods

### Subjects and experimental design

The Regional Scientific Ethical Committees of Copenhagen in Denmark approved this study (Ref: HD-2008-074), and all procedures conformed to the Declaration of Helsinki. The participants were all young healthy males who gave informed consent on inclusion. The muscle biopsies analysed in this study are a subset of samples collected during a larger study [12]. Briefly, volunteers were subjected to a muscle injury protocol which consisted of 200 forced lengthening contractions of the vastus lateralis muscles of one leg, with the use of electrical stimulation to initiate each contraction, as described in detail [12].

### Muscle sampling and preservation

Muscle biopsies were collected from the vastus lateralis muscle before the injury protocol and afterwards on days 2, 7 and 30. Biopsies were taken under local anaesthetic (1% lidocaine: Amgros I/S, Copenhagen, Denmark), using the percutaneous needle biopsy technique of Bergström [19], with 5–6-mm-diameter biopsy needles and manual suction. On extraction, portions of the biopsy were prepared for preservation in three ways: (1) a small part of the sample was immersed in 2% glutaraldehyde in 0.05 M sodium phosphate buffer (pH 7.2) for transmission electron microscopy (TEM). (2) Parts of the biopsy suited for histology were aligned in parallel, embedded in Tissue-Tek (Sakura Finetek Europe, Zoeterwoude, The Netherlands), frozen in isopentane, pre-cooled by liquid nitrogen and stored at − 80 °C until analysis. (3) If the biopsy contained long intact bundles of fibres, those were prepared for single fibre analysis [20]. Fibre bundles were pinned to maintain fibre length and covered in Krebs-Henseleit bicarbonate buffer (containing 0.1% procaine) for 2 min, followed by Zamboni fixative (2% formaldehyde, 0.15% picric acid) for 30 min. The fibres were then transferred to a glass jar containing fresh Zamboni fixative and placed in the fridge for approximately 4 h, after which the Zamboni was replaced by 50% glycerol in PBS. On the following day, the jar was moved to a − 20 °C freezer for storage. For the present study, 4 subjects were selected based on the availability of single fibres collected from the injured leg on day 7 and the control leg of the same individual, as well as evidence of necrosis on cross sections on day 7. The age, height and weight of these subjects was 23 ± 4 years, 1.79 ± 0.04 m and 69 ± 7 kg, respectively. Samples analysed by electron microscopy included 2 of these subjects and 3 additional subjects, based on availability of tissue.

### Immunostaining of single fibres

Single muscle fibres were teased from the fibre bundles in PBS with the aid of a stereomicroscope. For the samples collected on day 7, bundles of 2–3 fibres were isolated together due to the brittle nature of the damaged fibres at this time point. Fibres were transferred to wells (12-well Nunc plate) containing immunobuffer (IB:PBS containing 50 mM glycine, 0.25% BSA, 0.03% saponin, 0.05% sodium azide) and stained as described previously [21]. Primary antibodies were diluted in IB and added to the wells. The plate was subjected to gentle shaking overnight at room temperature. Triton (0.1%) was added with the primary antibody for staining with some antibodies. After washing in 3 changes of IB (15 min each), the fibres were incubated with the secondary antibodies for 2 h. Following 3 washes, nuclei were stained with 1 μg/ml Hoechst 33342 (H1399; Invitrogen A/S, Taastrup, Denmark) for 5 min. The fibres were washed twice and individually aligned in a drop of mounting medium (Molecular Probes Prolong Gold mounting, cat. no. P36930, Invitrogen) on a microscope slide, coverslipped and stored at − 20 °C until being imaged.

### Antibody combinations and analyses

Single fibres were stained with various combinations of the antibodies mouse anti-CD56 (cat. no. 347740, Becton Dickinson, San Jose, CA, USA), mouse anti-nestin (10c2, cat. no. sc-23927, Santa Cruz Biotechnology, Inc., Heidelberg, Germany), rabbit anti-Ki67 (cat. no. CP249, Biocare Medical, Concord, CA, USA), mouse anti-CD68 (cat. no. M0718, Dako Denmark A/S, Glostrup, Denmark), rabbit anti-desmin (cat. no. ab32362, Abcam, Cambridge, UK), rabbit anti-laminin (cat. no. Z0097, Dako Denmark A/S, Glostrup, Denmark), goat anti-collagen IV (cat. no. AB769, Merck Millipore, Darmstadt, Germany) and mouse anti-Pax7 (cat. no. Pax7, Developmental Studies Hybridoma Bank, Iowa, IA, USA). Molecular Probes Alexa Fluor 568-labelled phalloidin (cat. no. A12380, Invitrogen) was used to stain F-actin. Viewing the nuclear staining alone by widefield fluorescent microscopy, all fibres were evaluated for general fibre morphology and cellular infiltration (*n* = 4). For the measurement of regenerating zone dimensions, single fibres were stained for desmin, CD68 and collagen IV, followed by secondary antibodies Alexa Fluor donkey anti-rabbit 568 (cat. no. A-10042, Invitrogen), Alexa Fluor donkey anti-mouse 488 (cat. no. A-21202, Invitrogen) and Alexa Fluor donkey anti-goat 647 (cat. no. A-21447, Invitrogen). A minimum of 20 fibres from the injured and 20 from the control sample for each subject (*n* = 4) was stained. Stained single fibres were viewed by wide-field microscopy, and the length and diameter of regenerating and necrotic zones, as well as cell-type content, was determined. Confocal microscopy was performed to obtain more detailed insight. Confocal images were acquired with a Zeiss LSM710, using the following objectives: ×20/0.8 Plan-Apochromat, ×40/1.3 oil DIC EC Plan-Neofluar and ×63/1.4 oil DIC Plan-Apochromat. Hoechst, Alexa Fluor 488, Alexa Fluor 568 and Alexa Fluor 647 were excited by a 405-nm diode laser, a 488-nm argon laser, a 543-nm HeNe laser and a 633-nm HeNe laser, respectively.

### Immunostaining of frozen sections

Frozen tissue was sectioned at − 20 °C using a cryostat, and sections were placed on glass slides (Superfrost Plus), which were stored at − 80 °C before being stained. For the serial sectioning, 60 12-μm-thick sections were cut from day 7 biopsies obtained from the injured leg from the same 4 subjects used in the single fibre measurement of regenerating zones. Serial sections were also cut from a control biopsy from the same individual as a staining reference. Sections were stained with various combinations of antibodies against laminin, CD56, desmin and embryonic myosin (F1.652; Developmental Studies Hybridoma Bank); neonatal myosin (NCL-MHCn; Novocastra, Leica Microsystems A/S, Ballerup, Denmark); alpha-sarcoglycan (NCL-L-a-SARC, Novocastra); beta-dystroglycan (NCL-L-a-SARC, Novocastra); myogenin (F5d, Developmental Studies Hybridoma Bank); nestin, CD68, collagen IV and dystrophin (cat. no. D8168, Sigma-Aldrich Denmark A/S, Copenhagen, Denmark); myosin type I (BA.D5, Developmental Studies Hybridoma Bank) and myosin type II (A4.74, Developmental Studies Hybridoma Bank). The number of injured fibres 7 days post was determined from the sections stained with dystrophin, laminin and Pax7, where dystrophin-negative fibres were considered injured. Dystrophin-positive and dystrophin-negative (delineated by laminin) fibres were counted and the negative fibres were expressed relative to the total number of fibres. Injured fibres were also followed through the serial sections to confirm the changing fibre diameter and cell content observed in the single fibres.

### Transmission electron microscopy

Following 3 rinses in 0.15 M sodium phosphate buffer (pH 7.2), the specimens were postfixed in 1% OsO_4_ in 0.15 M sodium phosphate buffer (pH 7.2) for 2 h. After dehydration in a graded series of ethanol, the samples were transferred to propylene oxide and embedded in Epon (TAAB Laboratories Equipment Ltd., Aldermaston, UK). Ultrathin sections were cut with a Reichert-Jung Ultracut E microtome (Leica Microsystems), collected on 1-hole copper grids with formvar supporting membranes (Merck, Darmstadt, Germany) and stained with uranyl acetate and lead citrate. A Philips CM 100 transmission electron microscope (Philips, Amsterdam, The Netherlands) was used to view the sections, and digital images were obtained with an Olympus Soft Imaging Solutions (OSIS) Veleta side-mounted CCD camera (Olympus, Tokyo, Japan).

### Statistics

Data were analysed using version 7.0a of Prism for Mac OS X software (GraphPad Software, San Diego, CA, USA). The 4 zone diameter variables were compared by 1-way repeated measures ANOVA with Tukey’s post hoc test. The 2 zone length variables were compared by paired *t* test. Data are reported as means ± SD.

## Results

### Day 7 profiles of regenerating vs. control fibres

**Fig. 1:**
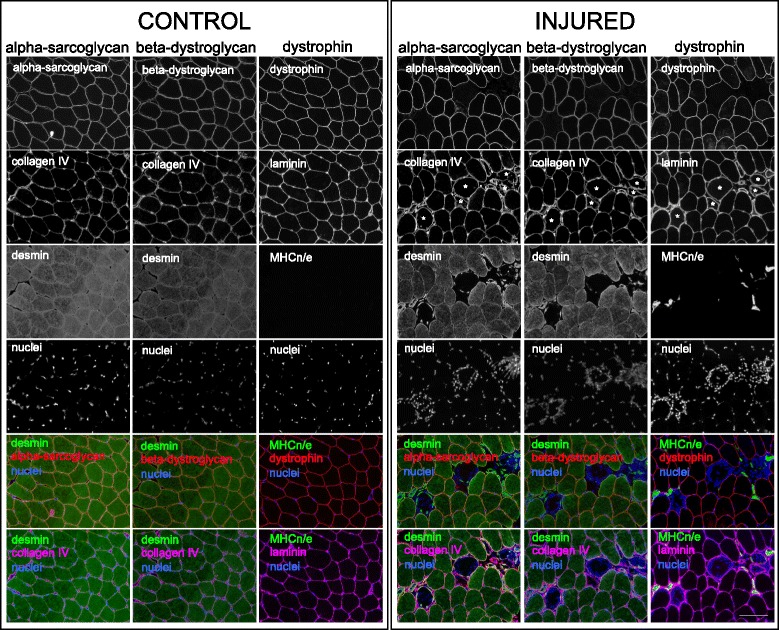
Cross-sectional profiles of regenerating fibres. Three serial frozen cross sections of a biopsy obtained from regenerating vastus lateralis skeletal muscle 7 days after injury induced by electrical stimulation-elicited eccentric contractions (right). Three serial cross sections from the control (uninjured) leg of the same individual are shown (left) for comparison. The sections have been stained with alpha-sarcoglycan, beta-dystroglycan or dystrophin to label the sarcolemma, along with a basement membrane protein (laminin or collagen IV) and a myogenic marker (desmin or neonatal/embryonic myosin; MHCn/e). Each column of images contains single channel and combined images for each staining. In the injured muscle, dystrophin staining is completely absent in several fibres, while the basement membrane (laminin) is preserved. MHCn/e staining is evident in some small dystrophin-negative fibres. A similar pattern is evident from the alpha-sarcoglycan and beta-dystroglycan staining, with negative fibres, together with desmin+ cells, contained within a preserved basement membrane (collagen IV). Asterisk indicates some of the necrotic fibres. Note the different profiles of the injured fibres, such as varying fibre size, and infiltrating cells either confined to the fibre periphery or dispersed throughout the fibre. Scale bar, 100 μm

From cross sections of regenerating muscle obtained 7 days post injury, different fibre profiles can be observed within the same biopsy specimen (Fig. 1). A mean of 26 ± 14% (range 9–42%) of fibres demonstrated a complete lack of dystrophin immunoreactivity, determined from 621 ± 372 fibres per biopsy cross section examined (*n* = 4), but with the preserved basement membrane components laminin and collagen type IV. No dystrophin-negative fibres were found in the control samples from the same subjects. A similar pattern was observed for alpha-sarcoglycan and beta-dystroglycan, with immunoreactivity for these proteins being absent in the same fibres where dystrophin was absent, again within a well-preserved basement membrane. In the injured leg, some of the dystrophin-negative fibres appeared to be swollen, with many nuclei at the fibre periphery. This was in stark contrast to the smaller fibres, which were densely packed with nuclei. Small compact patches of neonatal/embryonic myosin were also seen, but only at the fibre edge and typically only in the small fibres. To characterise these different profiles in more detail, several approaches were employed.

**Fig. 2:**
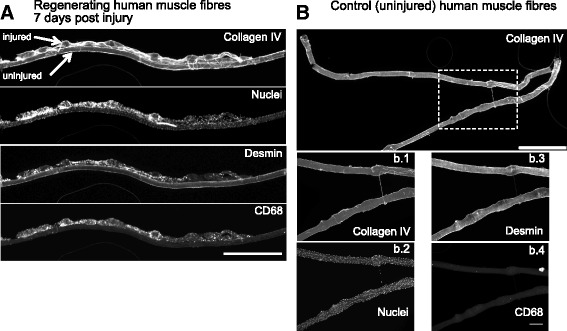
Longitudinal profiles of regenerating fibres. Wide-field microscope images of single muscle fibres used for the measurement of regenerating and necrotic zones, quantified in Fig. 3. Tissue was obtained from Bergström needle biopsies of regenerating (**a**) and control (**b**) human vastus lateralis muscles. Single fibres were stained for collagen IV, desmin, macrophages (CD68) and nuclei. The regenerating muscles contained both injured and uninjured fibres (**a**). The injured fibres were characterised by a repeating pattern of changing diameter along the length of the fibre, which contained macrophages and desmin+ cells within the basement membrane. Note the capillaries also stained with collagen IV. Scale bar, 1000 μm. In the control samples, rare fibres demonstrated regions of uneven fibre diameter (**b**, area in dashed line box magnified further in **b.1–4**) but did not contain macrophages or desmin+ cells. Scale bar, 1000 μm for **b**; 200 μm for **b.1–4**

The first was to evaluate the gross morphology of all stained single fibres from the injured leg 7 days post injury. One hundred thirty-three fibres in total were evaluated, where 41 ± 12% of fibres demonstrated a clear pattern of changing fibre diameter in a repeated manner along the length of the fibre and were densely filled with nuclei (see example in Fig. 2), with the remaining fibres appearing normal. The injured fibres were often found attached to completely unaffected fibres, as was seen on cross sections. Of the 46 single fibres isolated from a control sample, only 1 fibre demonstrated an uneven pattern of fibre diameter, although not to the same extent as in the injured leg and without the infiltration of clustered nuclei seen in the injured fibres.

**Fig. 3:**
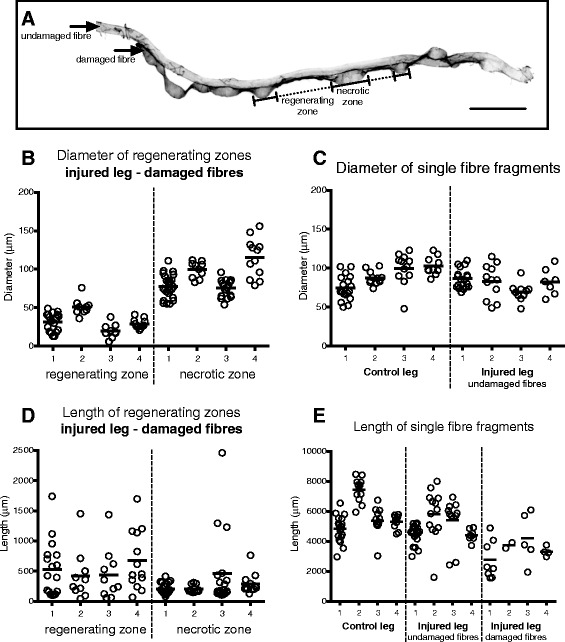
Single fibre zone dimensions. Dimensions of single muscle fibres obtained from control and regenerating human vastus lateralis muscle in 4 individuals (1–4), 7 days after injury. Individual data points with the mean are displayed. Within the damaged fibre (**a**), note the repeating pattern of regenerating (thinner) and necrotic (wider) zones (inverted wide-field immunofluorescent image of laminin staining, 500 μm scale bar). Within the damaged fibres, the diameter of the regenerating zones was significantly less than the diameter of the necrotic zones (**b**) and the control and undamaged fibres in the injured leg (**c**), *p* < 0.05. Displayed in **d** are the lengths of the regenerating and necrotic zones in the damaged fibres. The full length of the fibre fragments obtained during the isolation procedure is presented in **e**

To determine the dimensions and cell content of these fibres, single fibres stained for macrophages, desmin and basement membrane were studied more closely. Macrophages or desmin+ cells were not observed in any fibres from the control leg of the same individuals. The dimensions of the narrow cell-dense regenerating zones and the swollen necrotic zones seen in the injured muscle are presented in Fig. 3; 75 regenerating and 77 necrotic zones were measured. The diameter of the regenerating zones was consistently less than both the necrotic zones of the same fibres (*p* < 0.05) and the undamaged fibres from both the injured (*p* < 0.05) and control legs (*p* < 0.05) of the same individual. Within the damaged fibres, a greater variation was observed for the length of regenerating zones when compared to the necrotic zones; 100% of regenerating zones were observed to contain macrophages and 96% of these zones also contained desmin+ cells. Of the 77 necrotic zones measured, 97% contained macrophages, and 86% contained desmin+ cells (7 of these, all from the same individual, with very few desmin+ cells). All fibre fragments studied were either completely normal or regenerating (alternating necrotic and regenerating zones, with macrophage infiltration) along their entire length.

As shown in Fig. 3, the length of the fibre fragments ranged from 2966 to 8465 μm for the control leg samples and 1597 to 8001 μm for the undamaged fibres of the injured leg. The damaged fibres broke easily while being teased from the fibre bundles, so fewer of these were obtained and in general, they were shorter, ranging from 1550 to 6092 μm. It should be noted that the length of fibre fragments obtained from the fixed fibre bundles is determined by the length of the fibre bundles that are drawn into the barrel of the biopsy needle by suction at the time of biopsy sampling. In this study, fibre bundles were taken for the single fibre analysis only in large biopsies where long fibre bundles had been obtained.

### Day 7 confocal study of regenerating fibres

**Fig. 4:**
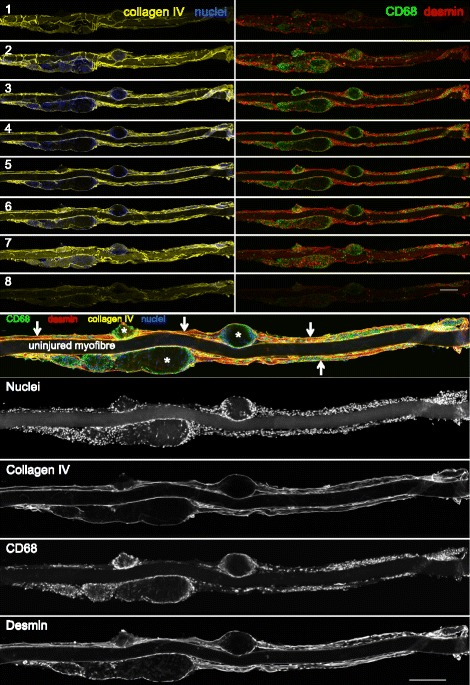
Detailed three-dimensional view of single fibres. Representative confocal microscopy images of single fibres from regenerating human skeletal muscle 7 days post injury. An intact uninjured fibre lies between 2 injured fibres. Eight individual slices (numbered) of a stack are displayed, with the 4 channels separated into basement membrane (collagen IV, yellow) and nuclei (blue), or macrophages (CD68, green) and desmin (red). Slice 5 is presented with 4 colours merged and single channel grey scale images below. The thinner regenerating zones (arrows) of the injured fibres contain macrophages and desmin+ cells and filaments, within the basement membrane. Both cell types are also present in the wider necrotic zones (asterisk) but only at the periphery (seen in slices 2 and 7), surrounding an acellular core (slices 4–5). Note macrophages in particular can be seen entirely cuffing the necrotic zones in the *x*, *y*, and *z* planes. Scale bars, 200 μm

**Fig. 5:**
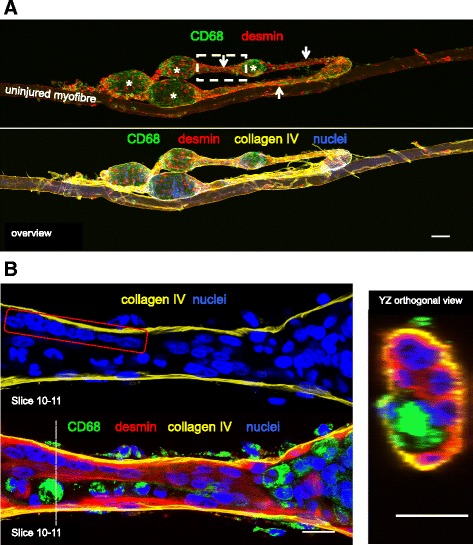
Myofilament formation at the basement membrane. **a**. Confocal microscope image of a regenerating human muscle fibre (attached to an uninjured fibre), stained for desmin (red), macrophages (CD68, green), basement membrane (collagen IV, yellow) and nuclei (blue), 7 days post injury. The images are presented with all 4 colours merged or split into fewer channels for clarity. Note the distinct necrotic (asterisk) and regenerating (arrows) zones. Part of the fibre has become detached from the uninjured fibre and folded back on itself during the staining procedure. Note the capillaries stained with collagen IV on the surface of the fibres. Scale bar, 100 μm. The area indicated by the dashed line box was imaged at higher magnification, shown in **b**. **b** Eight to nine adjoining nuclei can be seen (red box) in a desmin dense area of this regenerating zone, indicating they are potentially newly fused myogenic cells. Macrophages were also observed within the myofibre basement membrane in this narrow zone. The dashed line indicates the location of the YZ orthogonal view. Scale bars, 20 μm

**Fig. 6:**
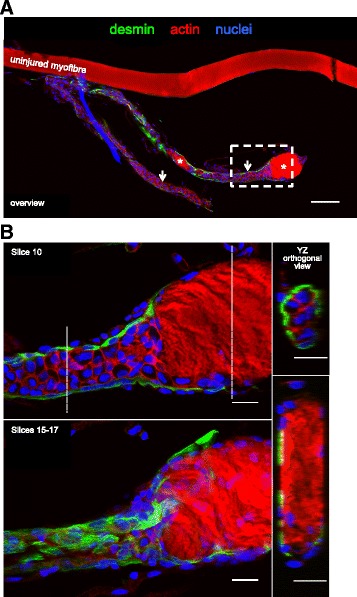
Lack of striations in necrotic zones. **a**. Low magnification confocal microscope image of a regenerating human muscle fibre 7 days post injury (and an uninjured fibre above it), stained for desmin (green), actin (phalloidin, red) and nuclei (blue). Note the distinct necrotic (asterisk) and regenerating (arrow) zones. Scale bar, 100 μm. The area indicated by the dashed line box was imaged at higher magnification, shown in **b**. The image slice (10) through the middle of the regenerating zone reveals a cell-dense area lacking actin striations. A desmin-positive sheath surrounds this region (slices 15–17). The dashed lines indicate the location of the two YZ orthogonal views. Note the wider necrotic zone devoid of cells and striations in its core. Scale bars, 20 μm

**Fig. 7:**
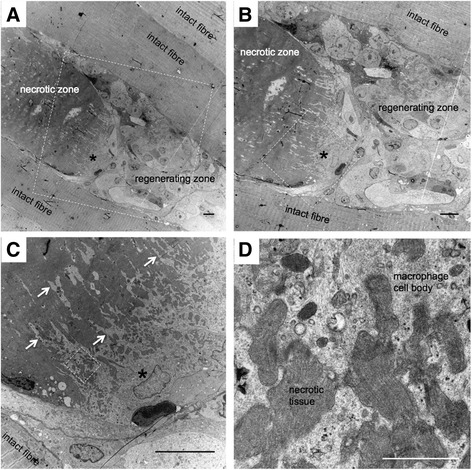
Macrophage pseudopodia infiltrate necrotic tissue. Transmission electron microscopy of biopsies obtained from regenerating human skeletal muscle 7 days post injury. **a** is the lowest magnification, with **b**–**d** increasing in magnification and the area shown outlined by the white dashed line box in the preceding image. Damaged fibres were observed adjacent to completely intact fibres (evident from the preserved striated sarcomere pattern clearly visible in the bottom left corner of image **c**). Within the damaged fibre, necrotic tissue can be seen to fill the wider zone and is followed by cell-dense regions. The asterisk (**a**–**c**) indicates the nucleus of a putative macrophage, situated at the mouth of the necrotic zone with its pseudopodia (white arrows) extending deep into the necrotic tissue (**c**, **d**). **a**–**c** Scale bars, 10 μm. **d** 1 μm

Confocal imaging of the immuno-stained single fibres revealed a continuous basement membrane defining the original muscle fibre (Figs. 4 and 5), within which mononuclear cells (macrophages, satellite cells and myoblasts), multinucleated myotubes and longer regions of immature sarcomeres were observed. Macrophages and desmin+ cells were observed together in the necrotic zones but only at the fibre periphery, surrounding a completely acellular core (Fig. 4). Macrophages were consistently seen to cuff the necrotic zones in all planes, often forming a clear boundary between the necrotic and regenerating zones (Fig. 4), as well as being dispersed throughout the regenerating zones to varying degrees, alongside desmin+ mononuclear and multinuclear cells (Figs. 4 and 5). Confocal analysis of the necrotic regions revealed a complete lack of sarcomere striations (Fig. 6). This was confirmed by TEM of longitudinally orientated tissue, where the classic sarcomere-banding pattern seen in adjacent uninjured fibres was absent in the injured fibres (Fig. 7). By TEM, multiple cell pseudopodia could be observed infiltrating the necrotic tissue from the mouth of the necrotic zone (Fig. 7). It is likely these are macrophages.

**Fig. 8:**
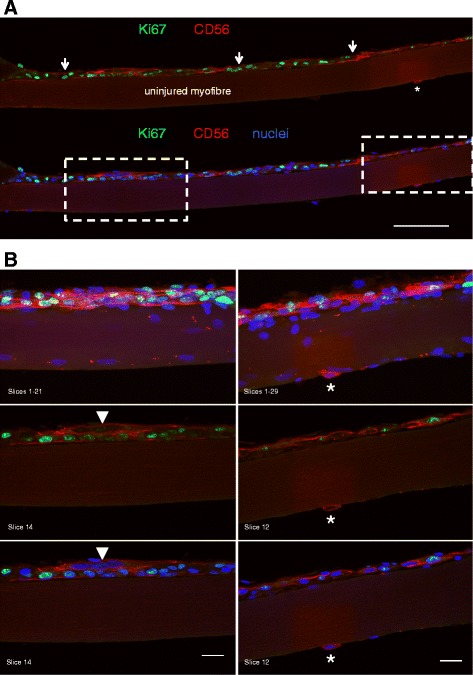
Proliferating cells and myotubes. Confocal images of a long regenerating zone (arrows) of a human skeletal muscle fibre, lying above an uninjured fibre, stained for the myogenic marker CD56 (red), cell proliferation marker Ki67 (green) and nuclei (blue). **a** scale bar, 100 μm. The areas indicated by the dashed line box were imaged at higher magnification, shown in **b**, where images are displayed as maximum intensity projections of multiple or single stack slices. CD56+ myogenic cells and myotubes (arrowhead) are visible. Asterisk indicates a single CD56+ satellite cell on the undamaged fibre. **b** Scale bars, 20 μm

**Fig. 9:**
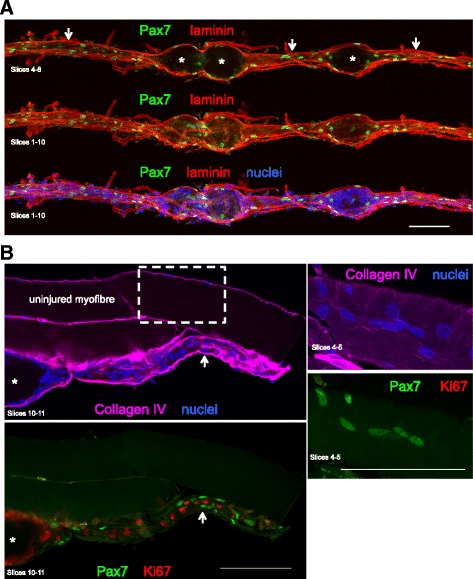
Pax7 staining. Confocal images of regenerating human skeletal muscle fibres. **a** A single muscle fibre is displayed, stained for laminin (red), Pax7 (green) and nuclei (blue), as maximum intensity projections of several slices to show that Pax7+ cells are located in both the regenerating zones (arrows) and at the periphery of the necrotic zones (asterisk). **b** Three fibres are visible, and the lowest fibre is undergoing regeneration. In the regenerating zone, it is clear that none of the Pax7+ cells is proliferating (Ki67+). The area indicated by the dashed line box is shown at higher magnification, where a cluster of Pax7+ cells was observed on the surface of the uninjured fibre. Scale bars, 100 μm

**Fig. 10:**
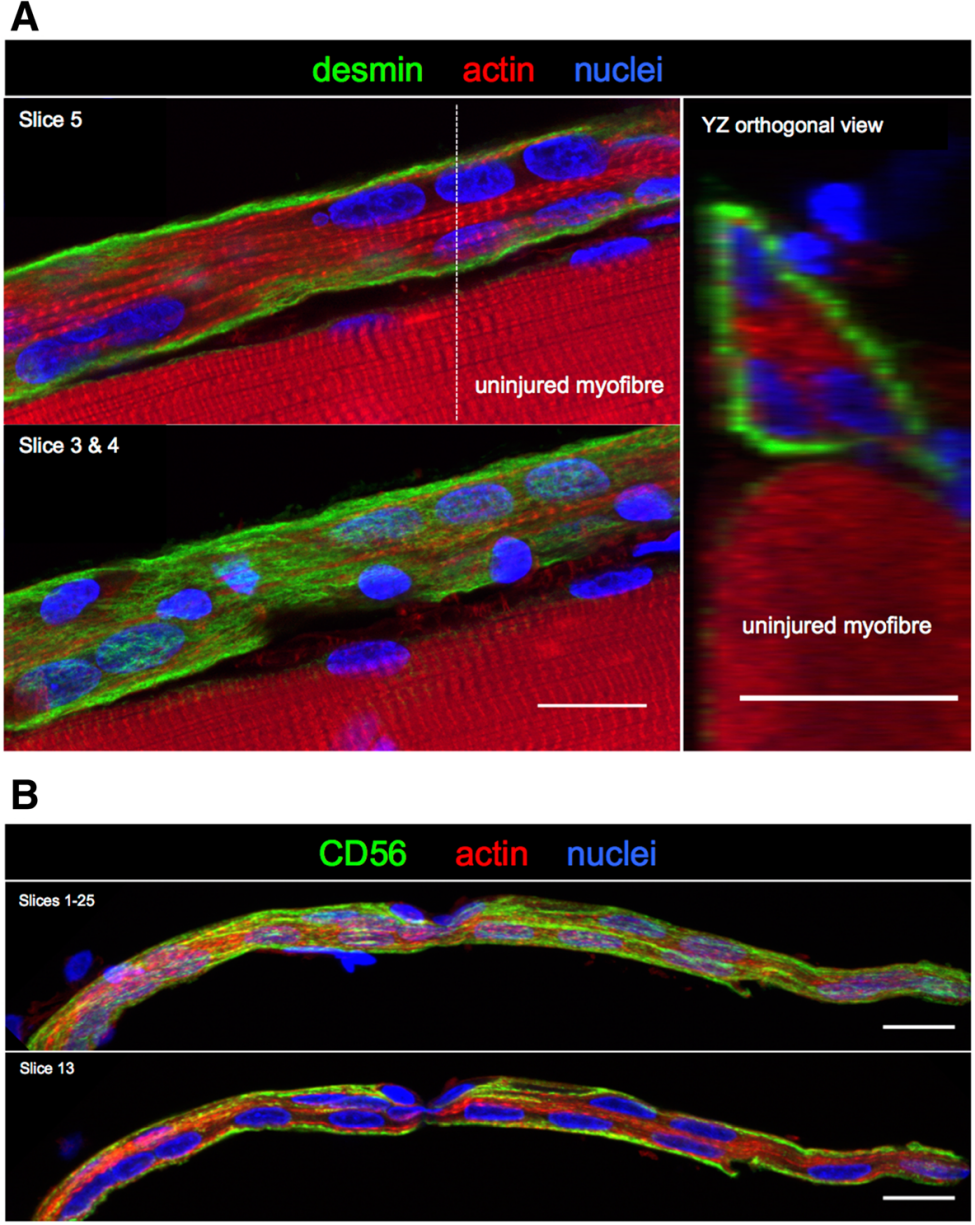
Early sarcomere formation. Confocal images of regenerating zones of human single muscle fibres at 7 days post injury, where immature actin striations (phalloidin, red) were clearly visible within a desmin+ (green) filamentous sheath (**a**) or within a CD56+ membrane (**b**). The nuclei (blue) are likely to be myonuclei. Note part of the adjacent undamaged fibre at the bottom of the image in **a**, where the mature striation pattern of the sarcomere can be seen. The dashed line indicates the location of the *YZ* orthogonal view. Scale bars, 20 μm

**Fig. 11:**
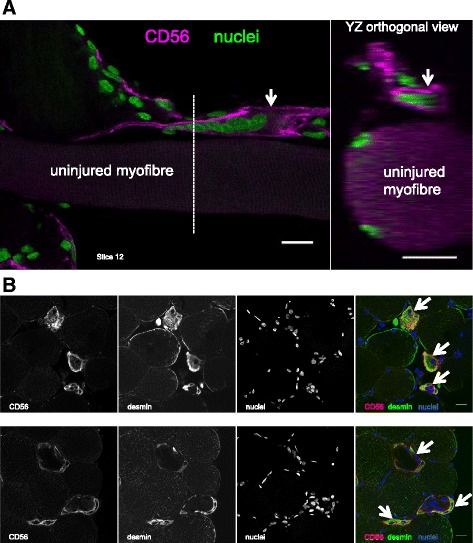
Myotube formation in vivo. **a** Single slice from a confocal stack of images of a regenerating zone of human skeletal muscle (day 7), capturing 6–7 adjoining nuclei (green) enclosed in a CD56+ (magenta) structure (arrow), likely a myotube, which is aligned along the fibre axis. Note the faintly visible adjoining uninjured fibre underneath, and part of another regenerating fibre at the bottom left of the image. The dashed line indicates the location of the *YZ* orthogonal view, where the fine CD56 staining can be seen entirely surrounding the myotube and where 2 peripherally located myonuclei are visible in the undamaged fibre. **b** Confocal images of 2 biopsy cross sections, from the same individual as in **a**, stained for CD56 and desmin, where CD56 can be seen encasing desmin+ structures, in 3 regenerating fibres (arrows) in each series of images. Scale bars, 20 μm

Within the narrow regenerating zones, two patterns were apparent, possibly representing distinct stages of myofibre regeneration. Either these zones were filled with cells (including myogenic Pax7+ or CD56+ cells), with no sign of sarcomere striations (Figs. 6, 8 and 9), or a clear striation pattern was evident in parallel with myonuclei aligned in series (Fig. 10). Strongly stained desmin+ cells were observed at the fibre periphery, either as mononuclear cells at the periphery of a necrotic zone (e.g. Fig. 4, slice 7) or as a formation of a continuous desmin+ network, encasing the narrow cell-dense zone. Figure 10 is an example of a regenerating zone containing an immature sarcomere striation pattern and what appear to be myonuclei, completely enveloped by a desmin+ coating. Similar arrangements of many aligned and adjoining nuclei were observed in many fibres studied. An example is shown in Fig. 5, where it can also be seen that these nuclei did not belong to macrophages, and in Fig. 11, where such a series of nuclei was seen completely enveloped by a CD56+ layer, further supporting the idea that they are indeed newly fused myogenic cells. Macrophages were clearly seen alongside these structures in most samples studied, as can be seen in Fig. 5. Incidentally, staining for Pax7+ cells was not consistent. Some fibres contained many Pax7 cells in contrast to a complete lack of Pax7+ cells in many fibre fragments. While this may represent an actual finding, this was not studied in a systematic manner in this paper and it is likely that enumeration of specific cell populations is best performed on fixed or frozen tissue cross sections, e.g. as we recently reported for satellite cells and fibroblasts [22].

### Day 7 cross-sectional profiles

**Fig. 12:**
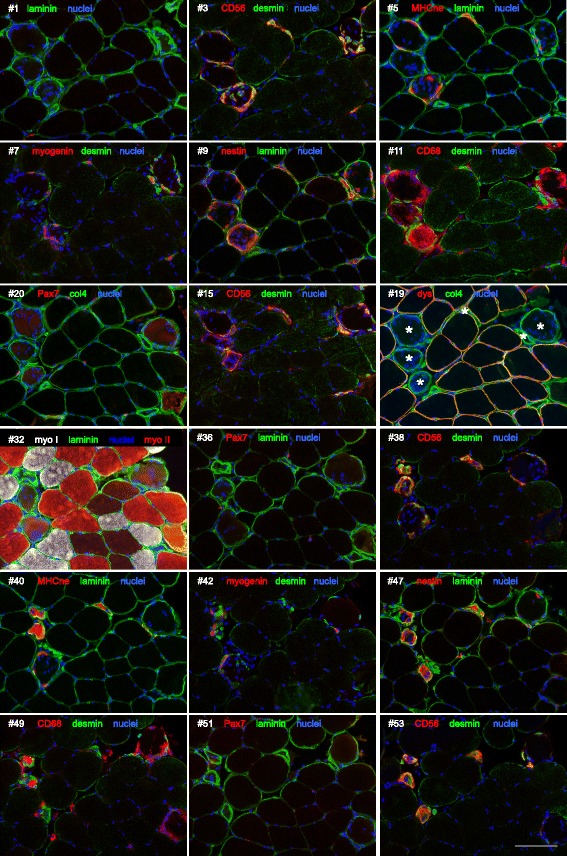
Myogenic and inflammatory cell activity viewed in serial cross sections. Microscope images of serial sections of one biopsy collected from regenerating human muscle 7 days after an injury protocol. Sixty serial sections (12 μm thick) were cut and stained for various markers. Shown here is a selection of these sections, where the section number (number sign) and proteins stained are indicated. Note the change in fibre diameter of the injured fibres, which are most clearly seen in section #19 (asterisk) by their lack of dystrophin (dys) staining and preserved basement membrane (collagen IV, col4). As observed in the single fibre analysis, these fibres are characterised by infiltration of macrophages (CD68+), the presence of neonatal/embryonic myosin (MHCne) and differentiating myogenic cells (desmin+, CD56+, myogenin+, nestin+). Scale bar 100 μm

To confirm that the presence of zones of varying diameter was not an artefact of the single fibre method (e.g. fibre hyper-contraction before fixation), serial sectioning of frozen biopsy material was performed. Many of the same features observed in the single fibres are also apparent in the injured fibres of these stained cross sections, such as changing fibre diameter and cell content, all within a preserved basement membrane (Fig. 12). Within these fibre scaffolds, cells positive for CD68 and a broad panel of myogenic markers (CD56, desmin, myogenin, nestin and neonatal and embryonic myosins) were observed.

### Day 30 features

**Fig. 13:**
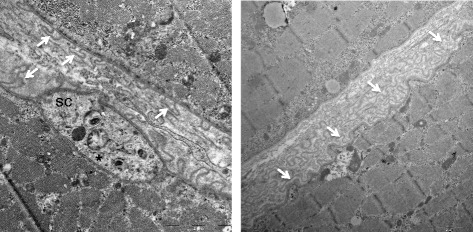
Original and new myofibre basement membrane. Transmission electron microscopy of biopsies obtained from regenerating human skeletal muscle 30 days post injury. Two basement membrane-like structures were observed around many regenerating muscle fibres. The outer basement membrane (arrows) consisted of empty pleats, which were clearly anchored to the muscle fibre and potentially are remnants of the original basement membrane. A continuous basement membrane, tightly associated with the sarcolemma as is seen in uninjured muscle, was also observed. Examples from 2 subjects are presented. A satellite cell (sc) is seen in the image to the left

**Fig. 14:**
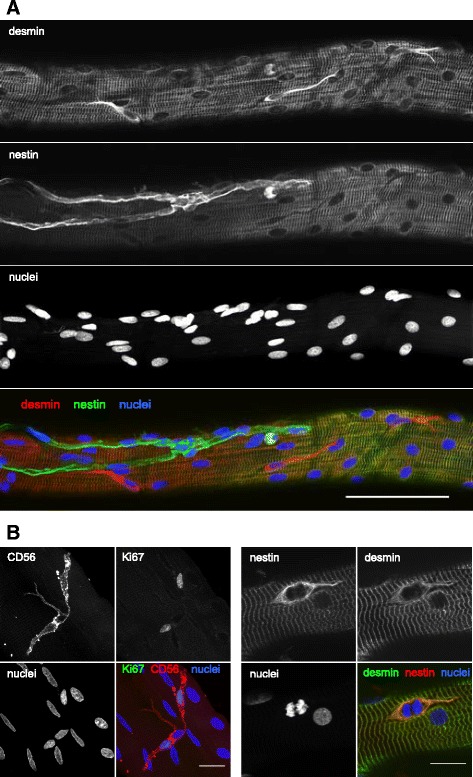
Satellite cells on single fibres. Confocal images of satellite cells on the surface of regenerating human muscle fibres 30 days post injury. Three desmin+ (nestin-negative) satellite cells are visible in **a**, where nestin demonstrates a striated staining pattern of the myofibre, indicating ongoing regeneration. Scale bar 100 μm. Proliferating satellite cells are shown in **b**, evident from the expression of Ki67 and CD56, or desmin and nestin together with a nucleus undergoing mitosis. Scale bars, 20 μm

Two basement membrane-like structures were observed by TEM around many regenerating muscle fibres at 30 days post injury in the 5 subjects studied. The inner basement membrane had the appearance of a continuous layer tightly following the sarcolemma, as in the uninjured samples. The second basement membrane structure had a pleated wrinkled appearance and consisted of empty loops, which were clearly anchored to the muscle fibre at both ends, as illustrated in Fig. 13. The muscle fibres themselves, while demonstrating a clear mature striation pattern at this time point, maintained some signs of on-going regeneration. These signs included the presence of nestin-positive striations and active satellite cells, the latter evidenced by strong desmin and nestin, or CD56 and Ki67 staining (Fig. 14).

## Discussion

In this first detailed study of regenerating adult human skeletal muscle, the main findings are that necrotic myofibre tissue is simultaneously removed and replaced within the same basement membrane scaffold, which is preserved throughout the continuum of muscle injury and repair until a new basement membrane is formed on the surface of the new myofibre. Myotubes form and fuse with each other, and myofibrillogenesis follows. As well as opening up a new method for following myogenesis in vivo in human skeletal muscle, these findings further illustrate the importance of the extracellular matrix in regulating myofibre repair.

**Fig. 15:**
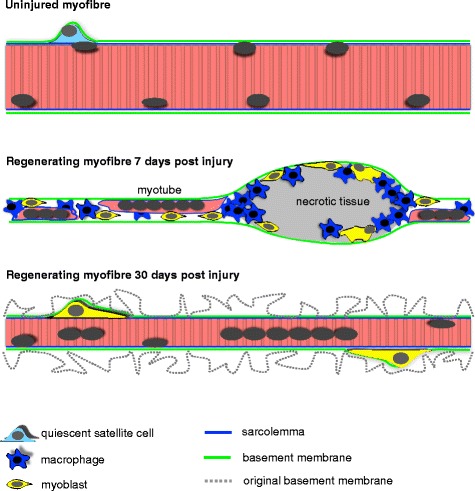
Overview of the regeneration of injured adult human skeletal myofibres. Upon injury, myofibres undergo necrosis, resulting in loss of the sarcolemma and removal of necrotic tissue by macrophages. The basement membrane, which is preserved, also acts as a scaffold for activated satellite cells to adhere to while they differentiate and fuse to form myotubes. Macrophages are also present in these zones. All the myotubes within a basement membrane tube eventually fuse with each other to form a single myofibre, which also forms a new basement membrane, resulting in a shedding of the original basement membrane. At 30 days post injury, the regenerating myofibres are still smaller than pre-injury and activated satellite cells are frequent on their surface

Based on our observations, in humans, the regeneration of skeletal muscle fibres that have been injured to the extent they undergo necrosis is characterised by the following events, as illustrated schematically in Fig. 15. Firstly, it appears that the electrically stimulated eccentric contraction model employed in our study induces complete fibre necrosis. Not all muscle fibres were affected, but in all the affected muscle fibres (26 ± 14%) we studied, we failed to find any regions demonstrating intact mature myofibre characteristics. Thus, our findings do not support the segmental model of muscle injury, which may be specific to focally induced injury [1, 2]. Rather, the present human model may have more in common with toxin-induced injury in the mouse, at least with regard to the extent of fibre injury. Secondly, the basement membrane around the necrotic fibres is preserved until the newly formed fibre forms a new basement membrane. This was apparent on single fibres, as well as cross sections, by the strong continuous staining of two major basement membrane components, laminin and collagen type IV. The lack of dystrophin staining around the damaged fibres indicates that sarcolemmal protein integrity has been severely compromised at this time point. This was supported by the lack of other sarcolemmal proteins, beta-dystroglycan and alpha-sarcoglycan (adhalin), as described previously for rodent muscle [23]. In addition to differences between adult satellite cells and embryonic founder stem cells, which form the first muscle fibres during embryonic development, as reviewed elsewhere [24], it would appear that the presence of a basement membrane template on which myogenesis can take place is a major discriminating feature between adult regenerative myogenesis and foetal myogenesis. Primary myotubes become apparent in developing mouse muscle at 12 days of development, while basement membrane formation does not appear until 18 days of development (2–3 days before birth), where the muscles begin to take on characteristics of mature muscle [25]. In the few studies of human foetal myogenesis, it seems that primary myotubes are apparent at 7 weeks [26], while basement membrane formation is evident by 14–15 weeks of development [27], clearly indicating that primary myotubes form without a basement membrane scaffold. In the finding of a “double” basement membrane 30 days post injury, it is likely that the second pleated basement membrane in fact represents the original basement membrane. At some point as the new myofibre is formed within this structure, it begins to form its own new basement membrane, and the process of shedding the original one begins. The double membranes seen in our samples are similar to those presented in older electron microscopy studies of animal muscle, where the importance of the basement membrane for successful regeneration was discussed [16–18]. It can be speculated that this process continues over several months until no remnants are visible and only the new basement membrane is visible. The implications of this are currently unknown, but in principle, this new basement membrane, together with the new sarcolemma, represents a completely new satellite cell niche, populated with a renewed pool of satellite cells.

The main event observed in our study was the replacement of a necrotic muscle fibre with an essentially new muscle fibre, formed through myogenesis. As the muscle fibre itself undergoes necrosis, macrophages infiltrate the dead tissue and begin the process of phagocytosis (Figs. 4 and 5). This occurs simultaneously at many sites along the length of the muscle fibre, rather than beginning at one end of the fibre. It is likely that the narrower regions observed to intersperse with the necrotic regions represent areas where the macrophages have already removed the necrotic tissue, and are thus at more advanced stage of regeneration. It can be speculated that this pattern is fully attributable to the 7-day time point at which the tissue samples were collected, rather than being specific to the electrical stimulation model employed in this study. Had we taken a muscle biopsy a few days after day 7, it can be speculated that the necrotic zones of the injured fibres would have all been removed, leaving only small fibres with the appearance of one long regenerating zone (as seen in Fig. 10). While this could be the case for muscle obtained from healthy volunteers, it is not known how diseased muscle would appear using this method. In particular, muscular dystrophy where a cycle of regeneration for one myofibre may never reach completion before the degeneration begins.

In some fibres, these regenerating zones contained only mononuclear macrophage and myogenic cells, while in others, multinucleated myotubes were observed, one of the hallmarks of myogenesis. It is interesting that both myogenic and macrophage-type cells were seen together in both zones, although myogenic cells and myotubes appeared to remain close to the basement membrane. While a major role of macrophages in this situation is in removing the necrotic tissue, the importance of macrophages in supporting myogenesis has clearly been shown, where pro-inflammatory and anti-inflammatory macrophages were found to stimulate myogenic cell proliferation and differentiation, respectively [28]. A high degree of cell proliferation was observed in the regenerating zones but did not appear to originate in myogenic cells, at least at the time point 7 days post injury. This was apparent from staining with the cell proliferation marker Ki67 together with the myogenic markers CD56 or Pax7. It is possible that the bulk of myogenic cell proliferation had already occurred at this time point, leaving macrophages as the most likely cell type capable of proliferating. This is supported by the presence of myogenin+ and desmin+ mononuclear cells, as well as neonatal and embryonic myosin, indicative of myogenic differentiation. Within the same basement membrane scaffold, many myotubes could be observed. CD56 was a reliable marker for myotubes, demonstrating a fine continuous staining of the myotube membrane, seen to typically contain 6–9 nuclei. Also, mono-nucleated CD56+ cells were seen in abundance in the regenerating fibres. While CD56 also labels nerves and natural killer cells, our co-staining of CD56 with desmin supports CD56 as a marker of myogenic cells and myotubes inside the regenerating fibre scaffolds. However, where we did not include desmin in the labelling experiments with CD56, we cannot rule out the possibility that some of the mono-nucleated CD56+ in the necrotic fibres could be non-myogenic. The observation of myotubes is interesting in that it not only illustrates clearly how muscle fibres are formed in the adult but also supports the explanation for the occurrence of so-called split or branched fibres [29–32], as simply the result of incomplete lateral fusion of myotubes during regeneration [9, 33]. If only one myofibre is to be formed within each basement membrane tube, all of the myotubes will have to fuse with each other, and incomplete fusion would lead to the appearance of split fibres. We did observe a few instances of this in our samples but the time course of sampling may not have been optimal to study this phenomenon. Further work employing this model of muscle injury in humans could include time points at 10–14 days to catch this process.

After the formation of myotubes, the next stage of myofibre formation observed was the presence of actin striations (phalloidin stained) in very small fibres. These striations represent sarcomere formation, albeit at an early stage of myofibrillogenesis. Desmin appeared as a filamentous sheath surrounding these fibres (Fig. 10), rather than a major component of the z-lines as seen in the uninjured fibres and also seen in the samples collected 30 days post injury. At this later stage, many satellite cells on regenerating fibres also stained strongly for desmin (Fig. 14). Cycling satellite cells 30 days post injury have been reported in regenerating mouse muscle [3], supporting our finding of on-going satellite cell activity at this time point. It is not known how long the process of muscle regeneration following such an injury stimulus takes to complete in humans, but it is clear that the main processes involved beyond 30 days are replacement of immature myosins by mature myosin types and growth of myofibres to their original size. The space occupied by the original myofibres (before injury) has been lost, so a strong growth stimulus will be required for these new fibres to regain their original space. In turn, this will also require major and continued remodelling of the muscle basement membrane and extracellular matrix surrounding these fibres. We and others have previously shown elevated gene expression levels of many components of the muscle extracellular matrix 4 weeks after a single bout of electrically stimulated or voluntary eccentric contractions [34, 35], supporting this idea of sustained matrix remodelling as fibres return to their pre-injury size. In a recent study of regenerating muscle at this time point, we observed 4 times baseline levels in the numbers of muscle fibroblasts, which can be explained by the prolonged matrix remodelling. However, in addition to their primary role as a source of matrix proteins, skeletal muscle fibroblasts also strongly stimulated myogenic precursor cell differentiation and fusion [22], uncovering a novel role for fibroblasts in contributing to the complete maturation of human myofibres undergoing regeneration after injury. Taken together, it seems that at our last sampling time point of 30 days post injury, in addition to the presence of striated myofibres with a new basement membrane, all the cells required for full restoration of the muscle are present and active, leading us to assume that the process will continue. Future work could examine muscle biopsies at 3–6 months post injury to determine the full length of time required for human muscle to regenerate and whether exercise and/or pharmacological interventions during this time help or hinder the process.

### Limitations and strengths of the method

In general, the single fibre method adds valuable information to the study of muscle regeneration that is lost in analysis of tissue cross sections. However, it is not without limitations. The fibres are subjected to many manipulations during the various stages of preparation, so the potential for artefacts is high. The fixation itself is also quite harsh, precluding the use of antibodies that perform better on, or even require, unfixed tissue (e.g. embryonic and neonatal myosins). In addition, it was clear from this study that fibres damaged by the electrical stimulation were more brittle and thus broke more easily while being teased from the fibre bundles, requiring the isolation of 2–3 fibres together, resulting in a selection of fibres that make to the microscopy stage. Lastly, we observed great variation in the pattern of Pax7 cell staining in particular on both intact and regenerating single fibres, ranging from dense clusters to more evenly spaced cells along the fibre, and even many fibre fragments completely lacking Pax7 cells. It is not clear if the endomysium and basement membrane of some fibre preparations can vary in permeability to antibodies or if this actually reflects the biology of these fibres. A lack of staining should therefore not necessarily be interpreted as a lack of protein, without validating the findings on serial sections. Nonetheless, despite these limitations, experience with the method together with the study of tissue sections (by light or electron microscopy) to control for preparation artefacts, as we did in this study, lends value to the single fibre method as a useful tool in studying muscle fibre dynamics in three dimensions, not just under conditions of regeneration but during any form of adaptation, as has been expertly demonstrated by others [20, 21, 36].

## Conclusions

In summary, this work is the first to provide evidence of the occurrence of myogenesis in vivo to replace whole necrotic myofibres in human adult skeletal muscle following experimental injury. The main advantage of this model is that all the biological components of the system are present, from the basic vascular and innervation aspects to the complete host of molecules and cell types comprising the myogenic stem cell niche. Through biopsy sampling, and potentially advanced in vivo imaging techniques, the steps of regeneration and activities of specific cell types can be followed as they occur in their native environment. A further advantage of this model is that the influence of various pharmacological agents on these processes can be investigated in an in vivo setting, which may prove useful in determining the applicability of findings originating in cell culture or animal models. In addition, confocal microscopy of regenerating single fibres can easily be applied to injury models in other species. Altogether, our study contributes to basic understanding of the process of muscle regeneration in humans and provides a strong case for further study of myogenic and non-myogenic cell activities as well as muscle matrix, in the context of healthy and diseased human skeletal muscle in addition to regenerative medicine.

## Abbreviations

IB: Immunobuffer
MHCn/e: Neonatal/embryonic myosin
PBS: Phosphate-buffered saline
TEM: Transmission electron microscopy

## References

[1] Papadimitriou JM, Robertson TA, Mitchell CA, Grounds MD (1990). The process of new plasmalemma formation in focally injured skeletal muscle fibers. J Struct Biol.

[2] Järvinen TA, Järvinen TL, Kääriäinen M, Kalimo H, Järvinen M (2005). Muscle injuries: biology and treatment. Am J Sports Med.

[3] Hardy D, Besnard A, Latil M, Jouvion G, Briand D, Thepenier C, Pascal Q, Guguin A, Gayraud-Morel B, Cavaillon JM (2016). Comparative study of injury models for studying muscle regeneration in mice. PLoS One.

[4] Lepper C, Partridge TA, Fan CM (2011). An absolute requirement for Pax7-positive satellite cells in acute injury-induced skeletal muscle regeneration. Development.

[5] Murphy MM, Lawson JA, Mathew SJ, Hutcheson DA, Kardon G (2011). Satellite cells, connective tissue fibroblasts and their interactions are crucial for muscle regeneration. Development.

[6] Yu JG, Malm C, Thornell LE (2002). Eccentric contractions leading to DOMS do not cause loss of desmin nor fibre necrosis in human muscle. Histochem Cell Biol.

[7] Crameri RM, Langberg H, Magnusson P, Jensen CH, Schroder HD, Olesen JL, Suetta C, Teisner B, Kjaer M (2004). Changes in satellite cells in human skeletal muscle after a single bout of high intensity exercise. J Physiol Lond.

[8] Thornell LE (2011). Sarcopenic obesity: satellite cells in the aging muscle. Curr Opin Clin Nutr Metab Care.

[9] Grounds MD (2014). The need to more precisely define aspects of skeletal muscle regeneration. Int J Biochem Cell Biol.

[10] Mackey AL, Kjaer M (2017). Connective tissue regeneration in skeletal muscle after eccentric contraction-induced injury. J Appl Physiol (1985).

[11] Crameri RM, Aagaard P, Qvortrup K, Langberg H, Olesen J, Kjaer M (2007). Myofibre damage in human skeletal muscle: effects of electrical stimulation vs voluntary contraction. J Physiol Lond.

[12] Mackey AL, Rasmussen LK, Kadi F, Schjerling P, Helmark IC, Ponsot E, Aagaard P, Durigan JL, Kjaer M (2016). Activation of satellite cells and the regeneration of human skeletal muscle are expedited by ingestion of nonsteroidal anti-inflammatory medication. FASEB J.

[13] Ciciliot S, Schiaffino S (2010). Regeneration of mammalian skeletal muscle. Basic mechanisms and clinical implications. Curr Pharm Des.

[14] Caldwell CJ, Mattey DL, Weller RO (1990). Role of the basement membrane in the regeneration of skeletal muscle. Neuropathol Appl Neurobiol.

[15] Cullen MJ, Mastaglia FL, Mastaglia FL, Walton J (1982). Pathological reactions of skeletal muscle. Skeletal muscle pathology.

[16] Vracko R, Benditt EP (1972). Basal lamina: the scaffold for orderly cell replacement. Observations on regeneration of injured skeletal muscle fibers and capillaries. J Cell Biol.

[17] Schmalbruch H (1976). The morphology of regeneration of skeletal muscles in the rat. Tissue Cell.

[18] Sanes JR, Marshall LM, McMahan UJ (1978). Reinnervation of muscle fiber basal lamina after removal of myofibers. Differentiation of regenerating axons at original synaptic sites. J Cell Biol.

[19] Bergstrom J (1975). Percutaneous needle biopsy of skeletal muscle in physiological and clinical research. Scand J Clin Lab Invest.

[20] Dahl R, Larsen S, Dohlmann TL, Qvortrup K, Helge JW, Dela F, Prats C (2015). Three-dimensional reconstruction of the human skeletal muscle mitochondrial network as a tool to assess mitochondrial content and structural organization. Acta Physiol (Oxf).

[21] Ralston E, Lu Z, Ploug T (1999). The organization of the Golgi complex and microtubules in skeletal muscle is fiber type-dependent. J Neurosci.

[22] Mackey AL, Magnan M, Chazaud B, Kjaer M (2017). Human skeletal muscle fibroblasts stimulate in vitro myogenesis and in vivo muscle regeneration. J Physiol.

[23] Vater R, Harris JB, Anderson VB, Roberds SL, Campbell KP, Cullen MJ (1995). The expression of dystrophin-associated glycoproteins during skeletal muscle degeneration and regeneration. An immunofluorescence study. J Neuropathol Exp Neurol.

[24] Tajbakhsh S (2009). Skeletal muscle stem cells in developmental versus regenerative myogenesis. J Intern Med.

[25] Ontell M, Kozeka K (1984). The organogenesis of murine striated muscle: a cytoarchitectural study. Am J Anat.

[26] MacIntosh BR, Gardiner PF, McComas AJ (2006). Muscle formation. Skeletal muscle form and function.

[27] Romero NB, Mezmezian M, Fidzianska A (2013). Main steps of skeletal muscle development in the human: morphological analysis and ultrastructural characteristics of developing human muscle. Handb Clin Neurol.

[28] Saclier M, Yacoub-Youssef H, Mackey AL, Arnold L, Ardjoune H, Magnan M, Sailhan F, Chelly J, Pavlath GK, Mounier R (2013). Differentially activated macrophages orchestrate myogenic precursor cell fate during human skeletal muscle regeneration. Stem Cells.

[29] Chan S, Head SI (2011). The role of branched fibres in the pathogenesis of Duchenne muscular dystrophy. Exp Physiol.

[30] Pichavant C, Burkholder TJ, Pavlath GK (2016). Decrease of myofiber branching via muscle-specific expression of the olfactory receptor mOR23 in dystrophic muscle leads to protection against mechanical stress. Skelet Muscle.

[31] Pichavant C, Pavlath GK (2014). Incidence and severity of myofiber branching with regeneration and aging. Skelet Muscle.

[32] Head SI (2010). Branched fibres in old dystrophic mdx muscle are associated with mechanical weakening of the sarcolemma, abnormal Ca2+ transients and a breakdown of Ca2+ homeostasis during fatigue. Exp Physiol.

[33] Grounds MD (2014). Therapies for sarcopenia and regeneration of old skeletal muscles: more a case of old tissue architecture than old stem cells. BioArchitecture.

[34] Mackey AL, Brandstetter S, Schjerling P, Bojsen-Moller J, Qvortrup K, Pedersen MM, Doessing S, Kjaer M, Magnusson SP, Langberg H (2011). Sequenced response of extracellular matrix deadhesion and fibrotic regulators after muscle damage is involved in protection against future injury in human skeletal muscle. FASEB J.

[35] Hyldahl RD, Nelson B, Xin L, Welling T, Groscost L, Hubal MJ, Chipkin S, Clarkson PM, Parcell AC (2015). Extracellular matrix remodeling and its contribution to protective adaptation following lengthening contractions in human muscle. FASEB J.

[36] Ralston E, Lu Z, Biscocho N, Soumaka E, Mavroidis M, Prats C, Lomo T, Capetanaki Y, Ploug T (2006). Blood vessels and desmin control the positioning of nuclei in skeletal muscle fibers. J Cell Physiol.

